# Burden and Determinants of Pressure Injuries in Adult Hospitalized Patients in Oman: A Multicenter Epidemiological Study

**DOI:** 10.3390/life16071088

**Published:** 2026-06-29

**Authors:** Fatma Al Maskari, Nasser Al-Salmi, Huda Al-Noumani, Maen Aljezawi, Eilean Rathinasamy Lazarus, Faisal Al Rashdi

**Affiliations:** 1 Department of Adult Health & Critical Care, College of Nursing, Sultan Qaboos University, Muscat 123, Oman; s46462@student.squ.edu.om (F.A.M.); hudasn@squ.edu.om (H.A.-N.); eilean@squ.edu.om (E.R.L.); 2 Princess Salma Faculty of Nursing, Al al Bayt University, Mafraq 25113, Jordan; maljezawi@aabu.edu.jo; 3 Department of Fundamentals and Administration, College of Nursing, Sultan Qaboos University, Muscat 123, Oman; f.alrashdi1@squ.edu.om

**Keywords:** pressure injuries, hospital-acquired pressure injuries, community-acquired pressure injuries, prevalence, risk factors, adult hospitalized patients, Oman

## Abstract

Background: Pressure injuries are a preventable source of morbidity, mortality, and excess healthcare costs among hospitalized adults. They represent a significant burden in acute and critical care settings in the Eastern Mediterranean region, yet data from Oman remain limited. This study aimed to determine the prevalence and burden of pressure injuries and to identify their clinical determinants among adult hospitalized patients in Oman. Methods: A multicenter, descriptive correlational cross-sectional study was conducted in four tertiary hospitals in Oman. A total of 169 adult inpatients were assessed using standardized pressure injury definitions and staging criteria. Point and periodic prevalence of pressure injuries, including hospital- and community-acquired cases, were calculated over a three-month data collection period. Demographic and clinical data (comorbidities, hemoglobin levels, prior pressure injury history, ventilator use) were extracted from medical records. Chi-square tests and multiple logistic regression were used to identify factors independently associated with the presence of pressure injuries. Results: The overall point prevalence of pressure injuries was 8.7%, including 4.2% hospital-acquired and 4.7% community-acquired cases; periodic prevalence over the three-month study period was also estimated to capture pressure injuries occurring at any time during hospitalization. Most lesions were located over bony prominences and ranged from stage 1 to stage 4. In multivariable analysis, lower hemoglobin levels (odds ratio [OR] 0.059, *p* = 0.019), prior pressure injury history (OR 0.156, *p* = 0.003), cancer diagnosis (OR 4.328, 95% CI: 1.225–15.291, *p* = 0.023), and ventilator use (OR 0.211, *p* = 0.001) were significantly associated with pressure injury development. Conclusions: Pressure injuries represent a considerable burden among adult hospitalized patients in Oman, with almost half of cases being hospital-acquired and therefore potentially preventable. Identified determinants particularly anemia, cancer, previous pressure injury, and mechanical ventilation highlight the need for targeted risk stratification and intensified preventive measures in high-risk groups. Integrating routine risk assessment, nutritional optimization, and device-related pressure relief into standard care may reduce pressure injury occurrence and improve patient outcomes.

## 1. Introduction

Pressure injury (PI) is defined as localized damage to the skin and/or underlying tissue caused by pressure or shear [1]. Most PIs occur over bony prominences, such as the sacrum, but they may also develop in mucosal tissues and in areas exposed to pressure from medical devices [2]. Hospital-acquired pressure injury (HAPI), also referred to as a pressure ulcer, bedsore, or decubitus ulcer, develops during hospitalization as a result of pressure or a combination of pressure, shear, and friction leading to tissue deformation or ischaemia [3,4,5]. PIs remain a major patient safety issue because they cause pain, prolong hospital stay, increase the risk of infection, and add substantial burden to healthcare systems. Globally, PI prevalence has been reported to range from 4% to 49% [6]. In the United States, approximately 1.33 million patients experienced pressure injuries in 2016 [7]. Among intensive care unit (ICU) patients, reported incidence rates include 5.9% in Korea, 11.2% in Japan, and 13.4% in Iran [7]. Although the Agency for Healthcare Research and Quality reported a 13% reduction in overall hospital-acquired conditions, HAPI incidence increased by 6% [8].

Beyond their clinical burden, PIs have important economic and psychosocial consequences. HAPIs are associated with prolonged hospitalization, greater use of healthcare resources, infection, pain, and legal costs [8]. They also negatively affect quality of life (QoL). Jackson et al. (2018) reported that patients living with PIs experience loss of mobility, independence, dignity, privacy, and social participation, which affects both personal and professional life [9]. These findings highlight the importance of patient-centered prevention and management strategies [9].

The development of PIs is influenced by both extrinsic and intrinsic factors. Extrinsic factors include pressure, moisture, friction, and shear [10], whereas intrinsic factors include advanced age, immobility, reduced activity, impaired sensory perception, malnutrition, anemia, poor tissue perfusion, and chronic diseases such as diabetes, cardiovascular disease, and cancer [3,7,8]. These risk factors are especially common in ICUs, surgical wards, and among critically ill, elderly, and malnourished patients [6,11,12]. International guidelines also emphasize that ICU patients are particularly vulnerable because of immobility, sedation, haemodynamic instability, and device-related pressure [1,8].

In the Middle East, PI prevalence continues to raise concern. A study from Sheikh Khalifa Medical City in Abu Dhabi showed that PI prevalence increased from 6.4% in 2013 to 10.4% in 2018, and the authors suggested that the actual rate might be even higher because of under-documentation [13]. In Oman, research has shown that PI prevention remains challenging. Al Shidi (2016) reported low levels of nurses’ knowledge regarding PI prevention and identified gaps in resources and policy implementation [14,15]. More recently, Aljezawi et al. (2024) reported a 21.8% prevalence of HAPIs in Omani ICUs, with the sacrum and stage II injuries being the most common [15]. Similarly, Al-Mamari et al. (2024) found that sacral PIs (64.1%) and stage II ulcers (73.1%) were predominant and identified ventilator use, cancer, and cerebrovascular accident as important risk factors [16]. These studies also highlighted gaps in prevention practices, with some high-risk patients not receiving adequate preventive care [15,16].

Despite increasing attention to PI prevention, there remains a limited body of multicenter evidence from Oman on PI prevalence and factors associated with the presence of pressure among adult hospitalized patients. Most local studies have focused on ICU settings or nurses’ knowledge and practices rather than broader inpatient populations [14,15,16]. Given the aging population and increasing burden of chronic illness in Oman, context-specific evidence is needed to inform prevention strategies, improve risk assessment, and strengthen clinical policies [17,18]. Therefore, this study was conducted to investigate the prevalence and associated factors of pressure injuries among adult hospitalized patients in Oman and to generate evidence that may support better prevention and improved patient outcomes.

## 2. Purpose of the Study

The purpose of this study was to generate context-specific evidence on the prevalence and factors associated with pressure injuries and their impact on quality of life among adult hospitalized patients in Oman.

## 3. Materials and Methods

### 3.1. Study Design

This study employed a multicenter, descriptive correlational cross-sectional design conducted in four tertiary hospitals in Oman. A multicenter, descriptive correlational cross-sectional design was used to estimate the point and periodic prevalence of pressure injuries and to examine associations between patient characteristics and the presence of pressure injuries among adult inpatients in four tertiary hospitals in Oman.

Point prevalence was defined as the proportion of adult inpatients who had at least one pressure injury on the survey day, calculated as the number of patients with at least one pressure injury divided by the total number of patients assessed on that day [19].

Periodic prevalence was defined as the proportion of adult inpatients admitted to the participating units during the three-month data collection period who had at least one pressure injury (hospital- or community-acquired) identified at any time during their hospitalization. Each patient was counted once, even if they had multiple pressure injuries or repeated assessments [20].

Because the study sample comprised all eligible inpatients present on predefined survey days, periodic prevalence in this study was operationalized as the proportion of patients in this sample who had a pressure injury at any time during the three-month period (i.e., number of patients with at least one pressure injury during the period divided by 169).

### 3.2. Study Population and Setting

This study was conducted in four tertiary government hospitals in Oman that provide comprehensive medical, surgical, and critical care services for adult patients. These hospitals are major referral centers in their respective regions and manage a wide variety of acute and chronic conditions, including patients with complex comorbidities and high-dependency care needs.

The study population comprised adult patients aged 18 years and above who were admitted to medical, surgical, and intensive care units during the data collection period. Eligible patients had been hospitalized for at least 24 h, were clinically stable enough to undergo a full skin assessment, and provided informed consent (or proxy consent when required). Patients admitted to pediatric, maternity, and psychiatric units, as well as day-case or short-stay units, were excluded, along with those who were critically unstable or had incomplete clinical records.

### 3.3. Sample Size and Sampling Technique

The sample size was calculated using the single-population proportion formula, assuming an expected pressure injury prevalence of 10% among adult hospitalized patients, a 95% confidence level, and a 5% margin of error. To compensate for potential non-response and incomplete data, 10% was added to the minimum required sample, yielding a target of approximately 165 participants; a final sample of 169 adult inpatients was included in the study, which met and slightly exceeded this requirement.

A consecutive sampling technique was used. On each predefined survey day, all adult patients who met the inclusion criteria and were present in the selected medical, surgical, and intensive care units of the four hospitals were screened and invited to participate. Eligible and consenting patients were enrolled until all accessible patients in the participating units had been assessed during the data collection period, ensuring that the sample reflected the routine case-mix of adult inpatients in these tertiary hospitals.

### 3.4. Instruments Used for Data Collection

Data were collected using four instruments: the World Health Organization Quality of Life Questionnaire (WHOQOL-BREF), a researcher-developed Prevalence and associated factors of Pressure Injury Tool, the Braden Scale, and the Norton Scale. These instruments were used to obtain data on patients’ quality of life, sociodemographic and clinical characteristics, pressure injury prevalence and associated factors of pressure injury risk status. Health-related quality of life was assessed using the WHOQOL-BREF in a subsample of participants who were clinically stable, cognitively able to respond, and able to communicate in Arabic or English. The WHOQOL-BREF was administered to all eligible patients with pressure injuries and to a comparison group of patients without pressure injuries who met the same eligibility criteria and were available during the data collection period. In total, 51 participants completed the WHOQOL-BREF. The WHOQOL-BREF is a 26-item questionnaire developed by the World Health Organization to assess quality of life across four domains: physical health, psychological health, social relationships, and environment [21]. Previous studies have demonstrated good reliability for the WHOQOL-BREF, with Cronbach’s alpha reported at 0.896 for the total scale, while domain reliabilities were generally above 0.70 except for the social relationship’s domain, which has sometimes been lower, such as 0.533 in one validation study [21,22]. Other studies in regional populations have reported overall Cronbach’s alpha values around 0.91, supporting its acceptable internal consistency [23]. In the current study, internal consistency of the WHOQOL-BREF was not recalculated; therefore, interpretation of the quality-of-life findings relies on previously reported reliability evidence for this instrument.

The researcher-developed Prevalence and associated factors of Pressure Injury Tool was used to collect sociodemographic data, clinical characteristics, laboratory values, comorbidities, pressure injury characteristics, and preventive interventions. Because this was a researcher-developed instrument, its validity and reliability depend on expert review and pilot testing rather than published psychometric studies.

The Braden Scale consists of six subscales: sensory perception, moisture, activity, mobility, nutrition, and friction/shear [24]. Previous studies have shown acceptable to good reliability, with Cronbach’s alpha ranging from 0.64 to 0.78 and high inter-rater reliability ranging from 0.946 to 0.964 using intraclass correlations [25]. Other reviews reported Cronbach’s alpha values ranging from 0.43 to 0.85 depending on the clinical setting and study population [24,25]. The Braden Scale was used in its standard clinical form, and reliability was not recalculated in this sample; interpretation therefore relies on previously published reliability evidence.

The Norton Scale assesses five domains, physical condition, mental state, activity, mobility, and incontinence and is also widely used in clinical practice for pressure injury risk assessment [26]. Evidence suggests that the Norton Scale has acceptable inter-rater reliability in clinical practice, although its predictive validity is generally described as low to moderate when compared with other pressure injury risk scales [27,28].

Pressure injuries were identified and staged according to the revised National Pressure Injury Advisory Panel classification system, which supports standardized assessment across settings [29]. All instruments were administered by trained data collectors using standardized procedures to improve consistency in data collection across the four participating hospitals.

### 3.5. Data Collection Procedure

Data were collected over a defined study period (July 2024 to January 2025) using a standardized protocol across the four participating tertiary hospitals. On each predefined survey day, the trained data collectors obtained the daily list of admitted adult patients from the nursing census in the medical, surgical, and intensive care units. Patients were screened against the inclusion and exclusion criteria, and eligible patients were approached, provided with information about the study, and invited to participate. Written informed consent was obtained from patients or their legal representatives before data collection commenced.

Following consent, a comprehensive head-to-toe skin assessment was performed at the bedside to identify and stage any pressure injuries using the revised National Pressure Injury Advisory Panel classification. For each participant, the researcher-developed data sheet was completed based on direct assessment and review of the electronic medical record, documenting sociodemographic details, clinical characteristics, comorbidities, laboratory values (including hemoglobin), history of pressure injury, PI characteristics, and preventive measures in place. Braden and Norton risk scores were recorded from the patient chart or assessed at the time of data collection according to unit practice. For patients with pressure injuries, the WHOQOL-BREF was administered to evaluate quality of life, either self-completed or interviewer-administered when necessary, due to physical limitations or literacy issues. All data collectors followed the same written instructions and were supervised during the initial phase of data collection to ensure consistency and adherence to the study protocol.

### 3.6. Data Management and Analysis

Data were entered, coded, and analyzed using SPSS version 27. Descriptive statistics (frequencies, percentages, means, and standard deviations) were used to summarize sociodemographic and clinical characteristics and to calculate point and periodic prevalence of hospital-acquired and community-acquired pressure injuries across the four hospitals. Point prevalence was defined as the proportion of adult inpatients who had at least one pressure injury on the survey day. Periodic prevalence was defined as the proportion of adult inpatients who had at least one hospital-acquired or community-acquired pressure injury identified at any time during the three-month data collection period. The WHOQOL-BREF domain scores (physical, psychological, social, and environmental) were computed and summarized descriptively. Normality of continuous variables, including WHOQOL-BREF items and domains, was assessed using the Kolmogorov–Smirnov and Shapiro–Wilk tests; significant deviations from normality supported the use of non-parametric approaches where appropriate.

Bivariate associations between pressure injury presence and potential associated factors (e.g., comorbidities, nutritional status, medication use, ventilator use) were examined using chi-square tests, and Spearman’s rank correlation was used for selected non-normally distributed variables. Variables that were statistically significant or clinically important were entered into a binary logistic regression model to examine independent associations with the presence of pressure injuries, adjusting for potential confounders. Adjusted odds ratios with 95% confidence intervals were reported, and statistical significance was set at *p* < 0.05. For the logistic regression, the dependent variable was the presence of at least one pressure injury (1 = pressure injury present, 0 = no pressure injury). Hemoglobin was entered as a continuous variable (per 1 g/dL increase). Categorical associated factors were coded as binary variables: prior history of pressure injury (1 = yes, 0 = no), ventilator use (1 = yes, 0 = no), and cancer comorbidity (1 = yes, 0 = no).

Given the small number of patients with pressure injuries (*n* = 15), the multivariable logistic regression model was restricted to a limited number of clinically and statistically important variables in order to minimize overfitting. Variables with strong bivariate associations (*p* < 0.05) and clear clinical relevance were considered for inclusion, and we ensured an acceptable events-per-variable ratio.

We examined the extent of missing data for key variables. Missingness was low/moderate (≤%) for most variables. Because the proportion of missing data was small and there was no clear pattern of differential missingness between groups, we performed a complete-case analysis for the main regression models. The number of observations used in each analysis is reported in the table footnotes.

Potential confounders were identified a priori based on clinical judgment and previous literature (e.g., age, number of comorbidities, BMI). In the bivariate analysis, variables associated with pressure injury presence at *p* < 0.05 and/or considered clinically important were considered for inclusion in the multivariable logistic regression model. Given the small number of patients with pressure injuries (*n* = 15), we limited the number of factors in the final model to reduce overfitting, prioritizing variables with strong bivariate associations and clear clinical relevance. Age, BMI, and comorbidity burden were retained as adjustment variables, while hemoglobin, prior pressure injury, cancer, and ventilator use were included as main predictors (Supplementary File S2).

### 3.7. Ethical Considerations

Ethical approval to conduct the study was obtained from the Institutional Review Board of the investigator’s affiliated university, College of Nursing, Sultan Qaboos University (CON/MSN/2024/9), as well as from the Health Studies and Research Approval Committee and the Ministry of Health, Sultanate of Oman, under proposal ID MoH/CSR/24/28441. Permission to use the WHOQOL-BREF was also obtained from the World Health Organization prior to data collection.

Written informed consent was obtained from all participants after providing a full explanation of the study purpose, procedures, potential risks and benefits, confidentiality measures, and the voluntary nature of participation. Information sheets and consent forms were provided in both English and Arabic, and participants were assured of their right to refuse or withdraw at any time without any effect on their care. Confidentiality and anonymity were maintained through the use of unique participant codes, removal of personal identifiers during analysis, and storage of all data in password-protected files accessible only to the research team. Data will be stored securely for five years and disseminated ethically through publications and conference presentations without revealing participants’ identities.

## 4. Results

### 4.1. Demographic, Clinical and Laboratory Characteristics

Table 1 presents the demographic, clinical, and laboratory characteristics of the 169 participants. The mean age was 62.8 years (SD 17.3), and more than half of the sample (57.4%) were aged 60 years or older; females slightly outnumbered males (51.5%). Most patients were admitted under medical units (63.9%), followed by critical care (23.7%), with a mean length of stay of 20.6 days (SD 22.7) and one-quarter hospitalized for more than 30 days. The mean BMI was 26.1 kg/m^2^ (SD 6.7), and over half of the patients were overweight or obese; nearly 60% had three or more comorbidities, most commonly hypertension (53.8%), diabetes (48.5%), and neurological disease (40.2). Almost half of the sample had hemoglobin levels below 10 g/dL, and hypoalbuminaemia was highly prevalent (82.5%), indicating substantial anemia and nutritional compromise in this cohort. More than one-quarter had a prior history of pressure injury, around one-fifth were receiving mechanical ventilation, and clinically important features such as incontinence-associated dermatitis (21.3%), edema (34.3%), delayed capillary refill (30.8%), and the use of sedatives, vasopressors, and steroids were frequent, reflecting a haemodynamically fragile, multi-morbid population at high risk for pressure injury development.

### 4.2. Prevalence of Pressure Injury

Among the 169 adult inpatients assessed, 15 had at least one pressure injury, corresponding to an overall point prevalence of 8.7% in the study population. Of these, 7 patients (4.2% of the total sample) had hospital-acquired pressure injuries and 8 patients (4.7%) had community-acquired pressure injuries, indicating that pressure injuries in this cohort arose at comparable rates before and during hospitalization.

**Figure 1:**
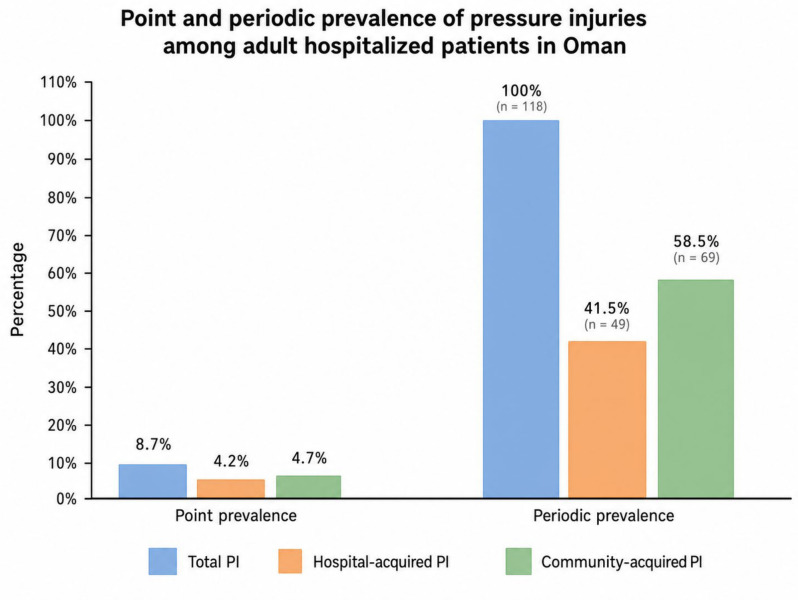
Point prevalence (patient-based) and cumulative lesion burden (lesion-based) of pressure injuries among adult hospitalized patients in Oman.

Figure 1 presents the point prevalence and cumulative lesion burden of pressure injuries among adult hospitalized patients in Oman. The overall point prevalence was 8.7%, with 15 of 169 inpatients having at least one pressure injury, including 4.2% hospital-acquired and 4.7% community-acquired cases. Over the three-month study period, a total of 118 individual pressure injury lesions were documented among these patients, of which 41.5% were hospital-acquired and 58.5% were community-acquired. This lesion-based estimate reflects the cumulative burden of pressure injuries rather than the proportion of patients affected and should therefore be interpreted separately from the point prevalence proportion.

#### Pressure Injury Characteristics

Table 2 summarizes the characteristics of pressure injuries among the 15 patients (8.9%) who had at least one PI out of the total sample of 169 inpatients. Of these lesions, 7 cases (4.2% of the total sample) were hospital-acquired and 8 cases (4.7%) were community-acquired, indicating that pressure injuries in this cohort occurred with comparable frequency before and during hospitalization. Most affected patients had a single ulcer (66.7%), while 33.3% had two or more lesions, reflecting a subgroup with more extensive skin breakdown and higher clinical complexity.

Anatomically, 9 of all recorded PIs (45.0%) were located over the sacrum/coccyx, followed by 5 heel injuries (25.0%), 3 trochanter/hip ulcers (15.0%), and 3 lesions (15.0%) at other bony prominences such as the occiput or malleoli, consistent with pressure concentration over weight-bearing areas in immobile patients. Stage 2 lesions constituted the largest group of deepest injuries (7 of 15; 46.7%), with additional cases classified as stage 1 (20.0%), stage 3 (20.0%), stage 4 (6.7%), and unstageable/deep tissue injury (6.7%), demonstrating a continuum from early to advanced tissue damage. Device-related PIs accounted for 3 cases (17.6%) among those with PIs, whereas 14 patients (82.4%) had non-device-related lesions, highlighting that while traditional pressure sites predominate, medical devices contribute to nearly one in five injuries in this sample.

Table 2 values describe individual pressure injury lesions. The total number of lesions exceeds the number of patients with pressure injuries (*n* = 15) because some patients had multiple lesions. Because some patients had more than one pressure injury, the lesion-level characteristics (e.g., anatomical location and stage) are based on the total number of pressure injury lesions rather than the number of patients.

### 4.3. Level of Risk for Pressure Injury

**Figure 2:**
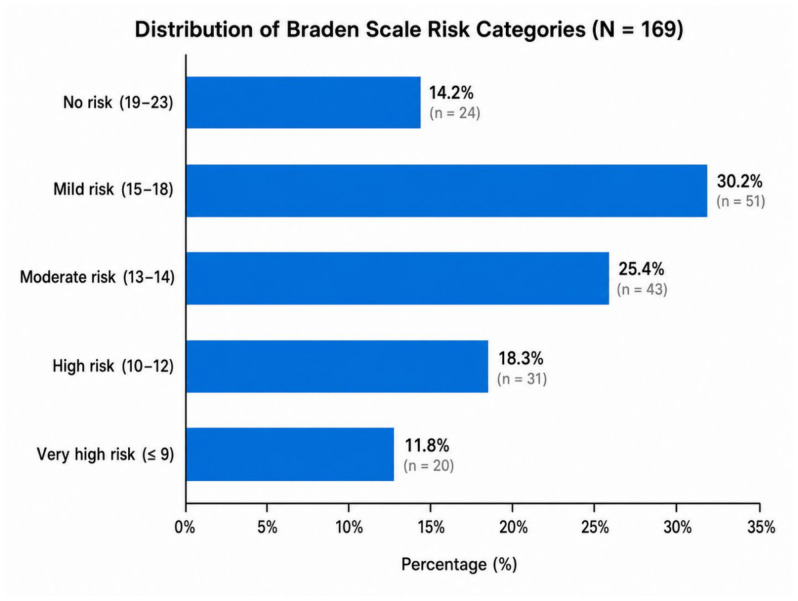
Distribution of Braden Scale Risk Categories (*n* = 169).

Figure 2 explains that most patients were classified as being at some level of risk for pressure injury on the Braden Scale, with only a small minority (around 14%) in the no-risk category. Approximately one-third (30.2%) were at mild risk, one-quarter (25.4%) at moderate risk, and nearly one-third (about 30%) fell into high or very high-risk categories (scores ≤ 12), indicating that a substantial proportion of the cohort required targeted preventive interventions according to standard Braden thresholds.

### 4.4. Preventive Measures for Pressure Injury

Table 3 describes the implementation of key preventive measures for pressure injury among the 169 hospitalized adults in the study. Regular repositioning at least every two hours and the use of pressure-redistributing mattresses were the most frequently documented interventions, applied to 84.0% and 81.1% of patients, respectively, reflecting good adherence to core recommendations from international guidelines on pressure injury prevention. Moisture management strategies (71.6%), routine skin inspection at least once per shift (79.9%), and the use of positioning pillows or wedges (69.8%) were also widely implemented, indicating that basic skin care and off-loading practices were generally integrated into routine nursing care.

However, some components of a comprehensive prevention bundle were less consistently applied. Nutritional support in the form of high-protein or high-calorie diets or supplements was provided to about two-thirds of the patients (66.3%), while physiotherapy or early mobilization was documented in just over half (52.7%), and patient or family education on pressure injury prevention in only 43.8%. This pattern suggests that although foundational measures such as repositioning and support surfaces are widely used, more proactive, multidisciplinary strategies including systematic mobilization and structured education may be underutilized, representing opportunities to strengthen prevention efforts in line with international best-practice recommendations.

### 4.5. Clinical and Risk Factors by Pressure Injury Status

Table 4 presents unadjusted comparisons of selected clinical and risk factors between patients with and without pressure injuries. Patients with PIs had a substantially longer hospital stay than those without PIs (mean 45.2 vs. 18.4 days; *p* < 0.001), suggesting that prolonged admission is strongly related to PI development in this cohort. Although the proportion of patients with three or more comorbidities was higher in the PI group (73.3% vs. 52.6%), this difference showed only a trend towards significance (*p* = 0.09).

Several factors differed significantly between the two groups. Patients with PIs more frequently had anemia, defined as hemoglobin below the reference threshold in g/dL (86.7% vs. 44.8%; *p* = 0.002), cancer comorbidity (40.0% vs. 11.0%; *p* = 0.004), ventilator use (66.7% vs. 17.5%; *p* < 0.001), and prior history of pressure injury (53.3% vs. 12.3%; *p* < 0.001). High or very high Braden risk scores (≤12) were also considerably more frequent among patients with PIs (73.3% vs. 26.0%; *p* < 0.001), as were incontinence-associated dermatitis (53.3% vs. 18.2%; *p* = 0.003) and edema (66.7% vs. 31.2%; *p* = 0.005), underscoring the combined contributions of multi-morbidity, anemia, malignancy, ventilator dependence, high risk status, moisture, and fluid imbalance to PI vulnerability prior to multivariable adjustment.

### 4.6. Associated Factors of Pressure Injury

The Table 5 explains the multivariable logistic regression model, hemoglobin level, prior history of pressure injury, cancer comorbidity, and ventilator use were independently associated with the presence of pressure injuries after adjustment for age, BMI, and comorbidity burden. In this sample, higher hemoglobin levels were associated with lower odds of having a pressure injury (adjusted OR 0.06, 95% CI 0.01–0.57, *p* = 0.019). A prior history of pressure injury was also associated with lower odds of having a pressure injury in the adjusted model (adjusted OR 0.16, 95% CI 0.05–0.49, *p* = 0.003), which is counterintuitive and should be interpreted cautiously in light of the small number of events and the possibility of residual confounding and coding effects. Cancer comorbidity was associated with higher odds of having a pressure injury (adjusted OR 4.33, 95% CI 1.23–15.29, *p* = 0.023). Ventilator use was associated with lower odds of having a pressure injury (adjusted OR 0.21, 95% CI 0.08–0.54, *p* = 0.001), again requiring cautious interpretation for the same reasons. Given the small number of patients with pressure injuries (*n* = 15), these associations should be considered exploratory and hypothesis-generating.

### 4.7. Health-Related Quality of Life (WHOQOL-BREF)

Health-related quality of life (WHOQOL-BREF) was evaluated in a subsample of 51 participants, including X patients with pressure injuries and Y patients without pressure injuries who met the eligibility criteria and were able to complete the questionnaire. Table 6 presents WHOQOL-BREF domain ratings for patients with and without pressure injuries. Only about one-fifth of participants in both groups reported good physical quality of life (Domain 1: 21.6% in controls vs. 21.4% in cases; *p* = 0.988), indicating that almost 80% perceived their physical health as poor regardless of pressure injury status. Psychological QoL (Domain 2) was also low overall, with 35.1% of patients without PI and 38.5% of those with PI reporting good psychological QoL (total 36.0%; *p* = 0.830). In contrast, social relationships (Domain 3) were rated more positively, with good QoL reported by 73.0% of controls and 64.3% of cases (70.6% overall; *p* = 0.543), and environmental QoL (Domain 4) was similarly favorable, with 73.0% of patients without PI and 57.1% of those with PI (68.6% overall) reporting good scores (*p* = 0.277). Thus, although none of the between-group differences reached statistical significance (all *p* > 0.05), the distribution of domain ratings suggests substantial physical and psychological burden in this cohort, alongside comparatively preserved social and environmental perceptions, a pattern consistent with previous reports that PIs and related chronic conditions primarily impair physical functioning and emotional well-being. The number of patients with pressure injuries (*n* = 15) was smaller than ideal for multivariable modeling and limits the events-per-variable ratio, so the regression findings should be interpreted as exploratory. Similarly, the WHOQOL-BREF subsample (*n* = 51) is likely underpowered to detect small or moderate differences between groups.

## 5. Discussion

This multicenter study examined the prevalence, factors associated with pressure injuries, preventive measures, and quality of life related to pressure injuries (PIs) among adult patients in four Omani acute-care hospitals [15]. The overall point prevalence of PI was 8.7%, including 4.2% hospital-acquired PIs (HAPIs) and 4.5% community-acquired PIs (CAPIs), indicating that PI burden arises both before and during hospitalization in this setting [15]. Point prevalence provides a snapshot of the proportion of inpatients with pressure injuries at a single time point, whereas the periodic measure captures the cumulative burden of pressure injuries over the study period. The latter is particularly useful for service planning but should be interpreted separately from the point prevalence proportion. These rates are comparable with international reports from general acute and medical–surgical units, where prevalence commonly ranges from 4% to 13% depending on case mix and methodology [9,30], but lower than findings from Omani critical care units, where HAPI prevalence has been reported at around 21.8%, reflecting the higher risk profile of ICU patients [31]. The predominance of sacral and heel injuries and the high proportion of stage II lesions are consistent with prior work and underscore the need to focus prevention on bony prominences exposed to sustained pressure and shear [1,32].

The demographic and clinical profile of the sample reflects a high-risk inpatient population. The mean age was 62.8 years and more than half of participants were aged 60 years or older, consistent with evidence that older adults are particularly vulnerable to PI due to age-related skin changes, reduced mobility, and multiple comorbidities [25,33]. Nearly 60% of participants had three or more chronic conditions, most commonly hypertension, diabetes, neurological disease, chronic kidney disease, and heart failure, and almost half had hemoglobin levels <10 g/dL with more than 80% showing albumin <35 g/L [34]. These findings mirror reviews that identify multi-morbidity, anemia, and hypoalbuminaemia as key contributors to impaired tissue tolerance and PI risk [35,36,37]. In addition, a substantial proportion of patients had a prior history of PIs, were mechanically ventilated, or showed clinical indicators such as edema, delayed capillary refill, incontinence-associated dermatitis, and exposure to sedatives, vasopressors, and steroids, all of which have been associated with increased PI risk in acute and critical-care cohorts [36,37,38,39].

Bivariate analyses showed that patients with PIs had longer hospital stays, indicating that prolonged admission was strongly associated with the presence of pressure injuries in this cohort, although the cross-sectional design does not allow conclusions about directionality [15]. These findings align with reviews and meta-analyses that have consistently identified anemia, hypoalbuminaemia, malignancy, immobility, moisture-associated skin damage, and a history of PIs as important risk factors [40,41,42]. The strong association between previous PIs and current lesions supports the concept of “tissue failure,” whereby previous damage and chronic microvascular compromise predispose patients to recurrent skin breakdown [1,43]. In the multivariable logistic regression, hemoglobin level, cancer comorbidity, ventilator use, and prior PI remained independent independently associated with the presence of pressure injuries [15]. The finding that lower hemoglobin increases the odds of PI is consistent with evidence that anemia reduces tissue oxygenation and impairs wound healing [42,44], while the association between cancer and PI echoes work showing that malignancy, systemic inflammation, cachexia, and treatment-related immunosuppression contribute to vulnerability to skin breakdown [40,41]. Ventilator use, a proxy for critical illness and immobility, has similarly been identified as strongly associated with hospital-acquired pressure injuries in previous studies [11,45,46]. Prior PI history remained a significant independent risk factor, highlighting the importance of aggressive secondary prevention strategies for patients with documented skin breakdown [1,43].

Regarding preventive measures, documentation indicated that most patients received core nursing interventions, including regular repositioning, pressure-relieving mattresses or overlays, heel protection, moisture management, and routine skin inspection [15]. These findings suggest that fundamental prevention strategies recommended by international guidelines are in place in the participating hospitals [1,47]. However, more proactive measures, such as early mobilization/physiotherapy, structured nutritional interventions, and formal patient/family education, were less consistently implemented [15]. Similar gaps between policy and practice have been reported in Omani ICUs and other settings, where not all high-risk patients receive appropriate support surfaces or repositioning regimes, and some low-risk patients receive unnecessary interventions [11,15]. These findings emphasize the need to move from reactive to anticipatory prevention, with systematic risk-based allocation of resources and auditing of adherence using key performance indicators embedded in institutional and national quality frameworks [1,14,16].

The WHOQOL-BREF data add an important patient-centered dimension. Although global ratings of QoL and health satisfaction did not differ significantly between patients with and without PIs, the domain-specific pattern was notable: 78.4% of the QoL subsample reported poor physical health and 64.0% reported poor psychological QoL, while 70.6% and 68.6% reported good social and environmental QoL, respectively [48]. These results mirror reviews and observational studies showing that PIs and chronic wounds primarily impair physical functioning and psychological well-being through pain, reduced mobility, fatigue, sleep disturbance, and distress whereas social and environmental domains are variably affected [49,50,51]. Item-level results, with particularly low ratings for energy, mobility, and opportunities for leisure, confirm that patients experience major limitations in daily living and participation even where they are relatively satisfied with living conditions, access to care, and social support [48]. Interestingly, the relatively favorable social and environmental QoL scores differ from several international studies in which PIs are associated with social withdrawal and environmental constraints [47,48,49]. In the Omani context, strong family networks and communal support may buffer the social impact of PIs, while access to tertiary care services and hospital infrastructure likely contributes to more positive environmental perceptions [15,31]. The lack of statistically significant differences in WHOQOL-BREF domains between cases and controls likely reflects the small QoL subsample, the high baseline burden of comorbidities in both groups, and the use of a generic rather than wound-specific QoL instrument, which may limit sensitivity to PI-specific effects [52,53].

Overall, the findings indicate that PIs remain a significant quality-of-care issue in Omani acute-care hospitals, particularly among older, anemic, multi-morbid patients and those with previous PIs, cancer, or ventilator dependence [32,54]. Clinically, these associated factors should be integrated into risk assessment and used to priorities high-risk patients for intensive preventive strategies, including nutritional optimization, early mobilization, rigorous skin and perfusion monitoring, and aggressive management of moisture and edema [55,56]. At the policy level, the results support reinforcing national PI prevention policies, standardizing the use of validated risk assessment tools, and institutionalizing PI surveillance with feedback to clinical teams [55]. From a patient-centered perspective, the WHOQOL-BREF findings highlight the need to integrate pain management, psychological support, rehabilitation focused on mobility and energy, and family-inclusive education into PI care bundles [7,49,54]. Future research in Oman should employ longitudinal designs and wound-specific QoL instruments in larger samples to better capture the causal impact of PIs on HRQoL and evaluate targeted interventions in high-risk subgroups, such as anemic, ventilated, or oncology patients with prior PIs [15,49,57,58,59].

Several methodological limitations should be considered when interpreting these findings. First, the cross-sectional design precludes establishing temporal relationships between patient characteristics and pressure injuries, and causal inferences cannot be drawn. Second, although we adjusted for selected confounders in the multivariable analysis, residual confounding from unmeasured or incompletely measured variables (e.g., detailed nutritional status, staffing patterns, or care processes) is likely. Third, measurement bias is possible because some clinical data, such as comorbidities and preventive interventions, were obtained from routine documentation, which may be incomplete or inconsistently recorded. Finally, selection bias may have occurred if patients who were too unstable or who declined participation differed systematically from those included in the study, potentially limiting the generalizability of the results. Taken together, these limitations indicate that the observed associations should be viewed as exploratory and hypothesis-generating, and confirmatory longitudinal studies are needed to better understand causal pathways and the impact of targeted prevention strategies.

We have structured the reporting of this observational study in line with the STROBE Statement, including detailed description of participant flow, variable selection, missing data handling, and limitations related to confounding and bias (Supplementary File S1).

## 6. Clinical Implications

This study identifies a clearly defined high-risk group. The factors found to be associated with pressure injuries in this cohort should be considered in routine risk assessment so that such patients are flagged early and prioritized for intensive prevention, including closer skin monitoring, more frequent repositioning, appropriate support surfaces, and careful management of moisture and edema. The high prevalence of anemia and hypoalbuminaemia highlights the need to integrate nutritional screening and early dietitian referral into PI prevention and care. For ventilated and critically ill patients, robust ICU-specific bundles combining repositioning, high-specification surfaces, and meticulous skin and device checks are essential.

Quality-of-life findings show major limitations in physical function, energy, and psychological well-being among patients with PIs, even when social and environmental support appear good. Clinical care should therefore extend beyond the wound itself to include pain control, rehabilitation focused on mobility and fatigue, and basic psychological support, with families actively involved in education and day-to-day preventive care. Finally, the persistence of PIs despite existing policies suggests that implementation is inconsistent. Units should regularly monitor PI rates, check whether high-risk patients are actually receiving the full prevention bundle, and use this information to guide staff education, resource allocation, and continuous quality improvement.

## 7. Strengths and Limitations

A key strength of this study is its multicenter design, which included adult patients from four tertiary hospitals and multiple clinical areas, providing a broad picture of pressure injuries in Omani acute-care settings. The study used standardized assessment tools and procedures (EPUAP methodology, Braden and Norton scales, WHOQOL-BREF), and combined detailed clinical data with both prevalence and predictive analyses. Collecting information on preventive measures and quality of life alongside clinical associated factor offers a more holistic understanding of PI burden than studies focused on a single outcome.

Several limitations should be acknowledged. The cross-sectional design does not allow causal inferences, and temporal relationships between patient characteristics and pressure injuries cannot be confirmed. The overall sample, and especially the 51-patient QoL subsample, was relatively small, limiting statistical power and making the absence of significant differences between groups potentially due to type II error; these findings should therefore be viewed as exploratory. The use of a generic QoL instrument rather than a wound-specific measure may have reduced sensitivity to pressure-injury-related effects. Some preventive and clinical data were taken from routine documentation, which may be incomplete or inconsistent and thus introduce measurement bias. Only 15 patients had pressure injuries, so the multivariable model was based on few events, increasing the risk of overfitting and wide confidence intervals; the regression results are therefore exploratory. For some instruments, formal reliability coefficients were not recalculated in this sample, which may limit the precision with which measurement error can be quantified. Finally, because the study was conducted in government tertiary hospitals, generalizability to private hospitals, smaller facilities, and community-based patients in Oman may be limited.

## 8. Conclusions

This multicenter study shows that pressure injuries are a significant problem among adult inpatients in four Omani acute-care hospitals, with an overall point prevalence of 8.7% and similar proportions of hospital- and community-acquired lesions. Patients were typically older, multi-morbid, anemic, and hospitalized for long periods, indicating a highly vulnerable population. Low hemoglobin, cancer comorbidity, ventilator use, and a prior history of PI were key factors associated with the presence of pressure injuries in this cohort, emphasizing the need to consider these characteristics in routine risk assessment and to prioritize affected patients for intensive preventive care. Although core preventive measures were often documented, more proactive components especially mobilization, nutrition, and structured patient/family education were not consistently applied. Quality-of-life findings revealed marked physical and psychological impairment, despite relatively good social and environmental support, highlighting that PIs affect daily functioning and emotional well-being as well as the skin. Overall, the results point to the need for stronger, risk-based prevention and more holistic, patient-centered PI care to improve outcomes for hospitalized adults in Oman.

## Figures and Tables

**Table 1:** Demographic, clinical, and laboratory characteristics of participants (*n* = 169).

Variable	Category	*n* (%)/Mean ± SD
Age (years)		62.8 ± 17.3
	18–39	30 (17.8)
	40–59	42 (24.9)
	≥60	97 (57.4)
Sex	Male	82 (48.8)
	Female	87 (51.5)
Marital status	Single	21 (12.4)
	Married	123 (72.8)
	Widowed/Divorced	25 (14.8)
Admitting unit	Medical	108 (63.9)
	Critical care	40 (23.7)
	Surgical	12 (7.1)
	Orthopedics	6 (3.6)
	Oncology	3 (1.8)
Length of stay (days)		20.6 ± 22.7
	≤7	49 (29.0)
	8–30	78 (46.2)
	>30	42 (24.9)
BMI (kg/m^2^)		26.1 ± 6.7
	Underweight (<18.5)	21 (12.4)
	Normal (18.5–24.9)	57 (33.7)
	Overweight (25–29.9)	40 (23.7)
	Obese (≥30)	51 (30.2)
Smoking status	Smoker	19 (11.2)
	Non-smoker	150 (88.8)
Number of comorbidities	0	13 (7.7)
	1–2	67 (39.6)
	3–4	73 (43.2)
	≥5	16 (9.5)
Key comorbidities	Diabetes mellitus	82 (48.5)
	Hypertension	91 (53.8)
	Chronic kidney disease	35 (20.7)
	Heart failure	35 (20.7)
	Neurological disease	68 (40.2)
	Cancer	23 (13.6)
	Respiratory disease	21 (12.4)
Hemoglobin (g/dL)		10.3 ± 2.1
	<10	82 (48.5)
	≥10	87 (51.5)
Albumin (g/L)	Abnormal (<35)	132 (82.5)
	Normal (35–60)	28 (17.5)
MAP category (mmHg)	Low (50–69)	18 (10.7)
	Normal (70–100)	136 (80.5)
	High (101–150)	15 (8.9)
SpO_2_ category	Normal (94–100%)	163 (96.4)
	Low (85–93%)	6 (3.6)
WBC category (×10^9^/L)	Abnormal low (<4)	5 (3.0)
	Normal (4–11)	95 (56.2)
	Abnormal high (>11)	68 (40.2)
History of pressure injury	Yes	47 (27.8)
	No	122 (72.2)
Ventilator use (current)	Yes	37 (21.9)
	No	132 (78.1)
Incontinence-associated dermatitis	Yes	36 (21.3)
	No	133 (78.7)
Capillary refill >2 s	Yes	52 (30.8)
	No	117 (69.2)
Presence of edema	Yes	58 (34.3)
	No	111 (65.7)
Medication use (selected)	Sedatives	64 (37.9)
	Vasopressors	39 (23.1)
	Steroids	47 (27.8)

**Table 2:** Pressure Injury Characteristics Among Participants with PI.

Variable	Category	*n* (%)
Presence of pressure injury	No PI	154 (91.1)
	≥1 PI	15 (8.9)
Type of PI	Hospital-acquired PI (HAPI)	7 (4.2)
	Community-acquired PI (CAPI)	8 (4.7)
Number of PIs per patient	1 lesion	10 (66.7)
	2 lesions	3 (20.0)
	≥3 lesions	2 (13.3)
Anatomical location (all PIs)	Sacrum/coccyx	9 (45.0)
	Heels	5 (25.0)
	Trochanter/hip	3 (15.0)
	Other bony prominences (e.g., occiput, malleolus)	3 (15.0)
Stage of deepest PI	Stage 1	3 (20.0)
	Stage 2	7 (46.7)
	Stage 3	3 (20.0)
	Stage 4	1 (6.7)
	Unstageable/deep tissue injury	1 (6.7)
Device-related PIs	Yes	3 (17.6)
	No	14 (82.4)

**Table 3:** Preventive Measures Implemented for Pressure Injury Risk (*n* = 169).

Preventive Measure	Implemented *n* (%)
Regular repositioning (at least every 2 h)	142 (84.0)
Use of pressure-redistributing mattress	137 (81.1)
Use of heel protectors/off-loading devices	96 (56.8)
Use of positioning pillows/wedges	118 (69.8)
Moisture management (barrier creams, incontinence care)	121 (71.6)
Skin inspection at least once per shift	135 (79.9)
Nutritional support (high-protein/high-calorie diet or supplements)	112 (66.3)
Physiotherapy/early mobilization	89 (52.7)
Patient/family education on PI prevention	74 (43.8)

**Table 4:** Bivariate Associations Between Patient Characteristics and Presence of Pressure Injury (*n* = 169).

Variable	No PI (*n* = 154) *n* (%)/Mean ± SD	PI present (*n* = 15) *n* (%)/Mean ± SD	*p* Value ^1^
Age (years), mean ± SD	62.3 ± 17.1	66.7 ± 18.4	0.32
Length of stay (days), mean ± SD	18.4 ± 20.1	45.2 ± 28.7	<0.001
Number of comorbidities ≥3	81 (52.6)	11 (73.3)	0.09
Hemoglobin < 10 g/dL	69 (44.8)	13 (86.7)	0.002
Cancer (Yes)	17 (11.0)	6 (40.0)	0.004
Ventilator use (Yes)	27 (17.5)	10 (66.7)	<0.001
Prior history of pressure injury (Yes)	19 (12.3)	8 (53.3)	<0.001
Braden high/very high risk (≤12)	40 (26.0)	11 (73.3)	<0.001
Incontinence-associated dermatitis (Yes)	28 (18.2)	8 (53.3)	0.003
Edema (Yes)	48 (31.2)	10 (66.7)	0.005

^1^ Chi-square or Fisher’s exact test for categorical variables; *t*-test or Mann–Whitney U test for continuous variables, as appropriate.

**Table 5:** Multivariable Logistic Regression: Predictors of Pressure Injury (*n* = 169).

Predictor	Adjusted OR	95% CI	*p* Value
Hemoglobin (per 1 g/dL ↑)	0.06	0.01–0.57	0.019
Prior history of PI (Yes vs. No)	0.16	0.05–0.49	0.003
Cancer (Yes vs. No)	4.33	1.23–15.29	0.023
Ventilator use (Yes vs. No)	0.21	0.08–0.54	0.001

**Table 6:** WHOQOL-BREF domain ratings in patients with and without pressure injuries (*n* = 51).

WHOQOL-BREF Domain	Patients Without PI (Controls, *n* = 37) % with Good QoL	Patients with PI (Cases, *n* = 14) % with Good QoL	Total (*n* = 51) % with Good QoL	*p* Value
Domain 1—Physical health	21.6	21.4	21.6	0.988
Domain 2—Psychological	35.1	38.5	36.0	0.830
Domain 3—Social relationships	73.0	64.3	70.6	0.543
Domain 4—Environment	73.0	57.1	68.6	0.277

## Data Availability

Data are available on reasonable request from the corresponding author. The datasets are not publicly available due to institutional and national regulations on patient data, but de-identified data may be shared for non-commercial research purposes subject to ethics committee and institutional approvals, and completion of a data-sharing agreement when required.

## Supplementary Materials

The following supporting information can be downloaded at: https://www.mdpi.com/article/10.3390/life16071088/s1, Supplementary File S1: STROBE Statement—Checklist of items that should be included in reports of cross-sectional studies; Supplementary File S2: Analytic procedures in IBM SPSS Statistics.

## Abbreviations

The following abbreviations are used in this manuscript:

PI	Pressure injury
HAPI	Hospital-acquired pressure injury
CAPI	Community-acquired pressure injury
QoL	Quality of life
WHOQOL BREF	World Health Organization Quality of Life—BREF
ICU	Intensive care unit
BMI	Body mass index
MAP	Mean arterial pressure
WBC	White blood cell count
IAD	Incontinence associated dermatitis
OR	Odds ratio
CI	Confidence interval
SD	Standard deviation
SPSS	Statistical Package for the Social Sciences
