# Supplementary material for: Burden and Determinants of Pressure Injuries in Adult Hospitalized Patients in Oman: A Multicenter Epidemiological Study
Source: Life (Basel). 2026 Jun 29;16(7):1088. doi: 10.3390/life16071088 (PMC13412731; doi:10.3390/life16071088)
Supplement: Supplementary file 1 [file life-16-01088-s001.zip › File S1 STROBE.pdf]

STROBE Statement—Checklist of items that should be included in reports of cross-sectional studies

Manuscript title: *Burden and Determinants of Pressure Injuries in Adult Hospitalized Patients in Oman: A Multicenter Epidemiological Study.*

Section / Topic	Item	Recommendation (short)	Where addressed in this manuscript
**TITLE & ABSTRACT**	1a	Indicate study design in title/abstract.	Title ("Multicenter Epidemiological Study") and Abstract – Methods ("multicenter, descriptive correlational cross-sectional study").
	1b	Informative, balanced summary of methods and findings.	Structured Abstract: Background, Methods, Results (prevalence, predictors), Conclusion.
**INTRODUCTION**	2	Background/rationale.	Introduction: paragraphs on global and regional PI burden, Middle East data, Omani context, gaps in prevalence/predictor evidence.
	3	Objectives/hypotheses.	"Purpose of the study" section: prevalence, predictors, QoL impact, and rationale for risk-based prevention strategies.
**METHODS**	4	Key elements of study design.	"Study Design" subsection: multicenter, descriptive correlational cross-sectional design in four tertiary hospitals.
	5	Setting, locations, dates, recruitment period.	"Study Population and Setting": four government tertiary hospitals, adult medical, surgical, ICU units, predefined survey days over a three-month data collection period.
	6a	Eligibility criteria, sources/methods of selection.	"Study Population and Setting" and "Sample Size and Sampling Technique": inclusion (≥18 years, ≥24 h stay, clinically stable, consent) and exclusion (pediatric, maternity, psychiatric, day-case, critically unstable, incomplete records); consecutive sampling on survey days.
	7	Definitions of outcomes, exposures, predictors, covariates.	"Instruments used for data collection" and "Data Management and Analysis": definitions of PI, HAPI, CAPI, point and periodic prevalence, Braden/Norton scores, comorbidities, haemoglobin, albumin, ventilator use, preventive measures, WHOQOL-BREF domains.
	8*	Data sources and measurement methods for each variable.	"Instruments used for data collection" and "Data Collection Procedure": bedside full-skin assessment using NPIAP classification, chart review for clinical/lab variables, recorded Braden/Norton scores, WHOQOL-BREF questionnaire for patients with PI.

	9	Efforts to address potential bias.	"Data Collection Procedure" and "Data Management and Analysis": trained data collectors, standardized protocol, multicenter sampling; "Discussion / Limitations" (if added) can note cross-sectional design, documentation quality, setting.
	10	How study size was determined.	"Sample Size and Sampling Technique": single-population proportion formula (expected 10% prevalence, 95% CI, 5% margin), plus 10% for non-response, resulting in target ~165 and final N = 169.
	11	Handling of quantitative variables (grouping, scores).	"Data Management and Analysis" and Table 1 footnotes: continuous variables summarized as mean ± SD; age, LOS, BMI, haemoglobin, albumin, MAP, WBC categorized; Braden risk categories; WHOQOL-BREF domains computed per manual.
	12a	Statistical methods, including confounding control.	"Data Management and Analysis": descriptive statistics, chi-square tests, Spearman's correlation, binary logistic regression with adjustment for age, BMI, comorbidity burden, and other clinically important predictors.
	12b	Methods for subgroup and interaction analyses.	"Clinical and Risk Factors by Pressure Injury Status" and "Predictors of Pressure Injury": bivariate comparisons PI vs no PI; multivariable model including several predictors simultaneously; no formal interaction terms reported.
	12c	Handling of missing data.	"Data Collection Procedure" and "Sample Size and Sampling Technique": exclusions for incomplete records at eligibility stage; main analyses based on available data (listwise deletion in regression).
	12d	Analytical methods accounting for sampling strategy.	"Sample Size and Sampling Technique" and "Data Management and Analysis": consecutive sampling of all eligible adult inpatients on survey days; no weighting applied.
	12e	Sensitivity analyses.	Not conducted; can be marked "Not applicable; no formal sensitivity analyses performed" in checklist.
**RESULTS**	13a	Numbers of individuals at each study stage.	"Demographic, clinical and laboratory characteristics" and "Prevalence of Pressure Injury": N = 169 included and analysed; description of hospital distribution and data-collection period (Figure 1, prevalence text).

	13b	Reasons for non-participation.	Explicit reasons for refusal not quantified; ineligibility criteria and exclusions (unstable, incomplete records, non-eligible units) described in Methods; note this in checklist as "Reasons for non-participation not systematically recorded."
	13c	Consider a flow diagram.	No flow diagram for participant inclusion; simple cross-sectional census; can be stated as "Not used."
	14a	Participant characteristics and key exposures/confounders.	"Demographic, clinical and laboratory characteristics" (Table 1); distributions of comorbidities, ventilator use, prior PI, IAD, oedema, medications, Braden risk; preventive measures (Table 3).
	14b	Numbers with missing data per variable.	Not reported per variable; Methods explain exclusion of incomplete records; note as "Not reported; incomplete records excluded at baseline."
	15*	Outcome events or summary measures.	"Prevalence of Pressure Injury," Figure 1, and Table 2: 15/169 with PI; 7 HAPI, 8 CAPI; periodic prevalence 118 cases (49 HAPI, 69 CAPI).
	16a	Unadjusted and adjusted estimates with precision; confounders specified.	"Clinical and Risk Factors by Pressure Injury Status" (Table 4: unadjusted comparisons with p-values) and "Predictors of Pressure Injury" (Table 5: adjusted ORs, 95% CI, p-values; adjustment for age, BMI, comorbidities).
	16b	Category boundaries when categorizing continuous variables.	Table 1 and Methods: age groups (18–39, 40–59, ≥60), LOS (≤7, 8–30, >30 days), BMI categories, haemoglobin <10 vs ≥10 g/dL, albumin <35 vs 35–60 g/L, MAP and WBC categories, Braden risk levels.
	16c	Translation of estimates into absolute risk.	Prevalence already expressed as absolute proportions; logistic estimates presented as OR only; no additional risk translation; may be marked "Not applicable."
	17	Other analyses (subgroups, interactions, sensitivity).	Descriptive analysis of preventive measures (Table 3); WHOQOL-BREF domain comparisons between PI and non-PI patients (Table 6 and related text); no formal interaction or sensitivity analyses.
**DISCUSSION**	18	Key results summary linked to objectives.	Discussion (not fully shown in excerpt, but described in thesis and Life draft): sections summarizing prevalence, predictors (haemoglobin, prior PI, cancer, ventilator use), preventive practices, and QoL patterns.

	19	Limitations, potential bias/imprecision, direction/magnitude.	“Discussion” and “Strengths and Limitations” (if retained from thesis): cross-sectional design, single time-point prevalence, relatively small PI case count, reliance on documentation, tertiary-hospital setting; direction (likely under-estimation of true burden).
	20	Overall interpretation considering objectives, limitations, and other evidence.	Discussion: comparison with regional and international prevalence and risk-factor studies; interpretation of anaemia, cancer, prior PI, ventilation as key determinants; implications for prevention, policy, and QoL.
	21	Generalisability (external validity).	Discussion / Implications: relevance to adult inpatients in Omani tertiary hospitals and similar Middle Eastern acute-care settings; limitations for smaller, rural, and private facilities.
**OTHER INFORMATION**	22	Funding and role of funders.	Not applicable
