# Supplementary material for: Burden and Determinants of Pressure Injuries in Adult Hospitalized Patients in Oman: A Multicenter Epidemiological Study
Source: Life (Basel). 2026 Jun 29;16(7):1088. doi: 10.3390/life16071088 (PMC13412731; doi:10.3390/life16071088)
Supplement: Supplementary file 1 [file life-16-01088-s001.zip › File S2. Analytic procedures in IBM SPSS Statistics.pdf]

File S2. Analytic procedures in IBM SPSS Statistics

All statistical analyses were conducted using IBM SPSS Statistics, version 27. Descriptive statistics (frequencies, percentages, means, and standard deviations) were generated for all sociodemographic, clinical, and laboratory variables, as well as for Braden risk categories, preventive measures, and WHOQOL-BREF domain scores. Categorical variables were summarized using frequencies and percentages, whereas continuous variables were summarized using means and standard deviations.

Prevalence calculations

Point and periodic prevalence of pressure injuries were calculated from SPSS frequency outputs. Point prevalence was defined as the proportion of adult inpatients who had at least one pressure injury on the survey day, computed by dividing the number of patients with one or more PIs (numerator) by the total number of inpatients assessed (denominator, N = 169) and multiplying by 100. Periodic prevalence was defined as the proportion of adult inpatients who had at least one hospital-acquired or community-acquired pressure injury identified at any time during the three-month data collection period, calculated using the total number of PI cases recorded over the period as the numerator and the total inpatient population during the same period as the denominator. The point and periodic prevalence values reported in the Results (text, Figure 1, and Table 2) were taken directly from these SPSS frequency tables.

Bivariate analyses

Bivariate associations between the presence of pressure injury (PI present vs PI absent) and potential risk factors (e.g., length of stay, comorbidity burden, anaemia, cancer, ventilator use, prior PI, Braden risk level, incontinence-associated dermatitis, oedema) were examined using the Crosstabs and Compare Means procedures. For categorical predictors, Crosstabs (Analyze → Descriptive Statistics → Crosstabs) was used with the 'Chi-square' option selected to obtain Pearson chi-square statistics, Fisher's exact test where appropriate, and row percentages. For continuous variables (e.g., length of stay), normality was inspected and group differences were evaluated using independent-samples t-tests or Mann–Whitney U tests as appropriate. The unadjusted p values presented in Table 4 and described in the Results section were copied directly from these SPSS outputs.

Multivariable logistic regression

Independent predictors of pressure injury were examined using binary logistic regression (Analyze → Regression → Binary Logistic). The dependent variable was PI status (1 = PI present, 0 = no PI). Predictor variables entered into the model were selected based on clinical relevance and significant bivariate associations and included haemoglobin level, prior history of pressure injury, cancer comorbidity, and ventilator use, with age, body mass index, and comorbidity burden considered as potential confounders. The 'Enter' method was used, and under 'Statistics' the options for model summary, classification statistics, Hosmer–Lemeshow goodness-of-fit, and 95% confidence intervals for Exp(B) were requested.

The logistic regression output provided unstandardized coefficients (B), standard errors, Wald statistics, odds ratios (Exp(B)), 95% confidence intervals, and p values. The adjusted odds ratios and confidence intervals reported in Table 5 were transcribed directly from the SPSS 'Variables in the Equation' table, and overall model fit indices were taken from the 'Model Summary' and Hosmer–Lemeshow tables. Manual checks were performed to ensure that all regression estimates in the manuscript matched the original SPSS output.

Quality of life analysis

For patients with pressure injuries and their matched controls, WHOQOL-BREF item scores were scored and domains (physical, psychological, social, environment) were computed according to the World Health Organization scoring guidelines. Domain scores were summarized descriptively and categorized into "good" versus "poor" quality of life according to established cut-offs. Group differences in WHOQOL-BREF domains between patients with and without PI were examined using chi-square tests for categorical classifications and non-parametric tests for continuous domain scores when appropriate, given the non-normal distribution identified in normality testing. The percentages and p values presented in Table 6 were copied directly from the SPSS output.

No additional data transformations were undertaken between SPSS output and table preparation other than formatting of categories and labels for presentation. All prevalence estimates, test statistics, and regression coefficients in the manuscript were cross-checked against the saved SPSS output prior to submission.
